# Reviewer acknowledgement 2014

**DOI:** 10.1186/s40337-015-0039-1

**Published:** 2015-03-17

**Authors:** Phillipa Hay, Stephen Touyz

**Affiliations:** School of Medicine, Centre for Health Research, University of Western Sydney, Sydney, Australia; School of Medicine, James Cook University, Townsville, Australia; Schools of Psychology and Centre for Eating and Dieting Disorders (Boden Institute), University of Sydney, Sydney, Australia

## Abstract

The *Journal of Eating Disorders* editorial team would like to thank all reviewers who have contributed to the journal in 2014.

## Reviewer acknowledgement

**Karina Allen**

Australia

**Jon Arcelus**

United Kingdom

**Rachel Bachner-Melman**

Israel

**Bryony Bamford**

United Kingdom

**Caroline Braet**

Belgium

**Cynthia Bulik**

United States of America

**Susan Byrne**

Australia

**Terry Carney**

Australia

**Louis Charland**

Canada

**Angelica Claudino**

Brazil

**Carolyn Costin**

United States of America

**Ross Crosby**

United States of America

**Scott Crow**

United States of America

**Lisa Dawson**

Australia

**Phillippa Diedrichs**

United Kingdom

**Murray Drummond**

Australia

**Pravin Dullur**

Australia

**Kamryn Eddy**

United States of America

**Sarah Fogarty**

Australia

**Andrea Goldschmidt**

United States of America

**Scott Griffiths**

Australia

**Susan Hart**

Australia

**Phillipa Hay**

Australia

**Natasha Hepworth**

Australia

**Gabriella Heruc**

Australia

**Hans W. Hoek**

Netherlands

**David Jimerson**

United States of America

**Allison Kelly**

Canada

**Janet Latner**

United States of America

**Sau Fong Leung**

Hong Kong

**Katharine Loeb**

United States of America

**Sarah Maguire**

Australia

**Barbara Mangweth-Matzek**

Austria

**Jeffrey Moss**

United States of America

**Richard Newton**

Australia

**Greta Noordenbos**

Netherlands

**Kenneth Nunn**

Australia

**Susan Paxton**

Australia

**Janette Perz**

Australia

**Kathleen Pike**

United States of America

**Kevin Pile**

Australia

**Janet Polivy**

Canada

**Cecile Rausch Herscovici**

Argentina

**Deborah Lynn Reas**

Norway

**Paul Rhodes**

Australia

**Lina Ricciardelli**

Australia

**Amanda Salis**

Australia

**Ulrike Schmidt**

United Kingdom

**Ulrich Schweiger**

Germany

**Lucy Serpell**

United Kingdom

**Pratap Sharan**

India

**Amy Slater**

United Kingdom

**Frederique Smink**

Netherlands

**Daniel Stein**

Israel

**Lois Surgenor**

New Zealand

**Amy Talbot**

Australia

**Marika Tiggemann**

Australia

**Stephen Touyz**

Australia

**Eva Trujillo**

Mexico

**Frederique Van den Eynde**

Canada

**Eric van Furth**

Netherlands

**Betteke van Noort**

Germany

**Tracey Wade**

Australia

**Cortney Warren**

United States of America

**Hunna Watson**

Australia

**Lucene Wisniewski**

United States of America

**Blake Woodside**

Canada

